# “How I Wish This Thing Was Initiated 100 Years Ago!” Willingness to Take Daily Oral Pre-Exposure Prophylaxis among Men Who Have Sex with Men in Kenya

**DOI:** 10.1371/journal.pone.0151716

**Published:** 2016-04-13

**Authors:** Robinson Njoroge Karuga, Serah Nduta Njenga, Rueben Mulwa, Nduku Kilonzo, Prince Bahati, Kevin O’reilley, Lawrence Gelmon, Stephen Mbaabu, Charles Wachihi, George Githuka, Michael Kiragu

**Affiliations:** 1 LVCT Health, Nairobi, Kenya; 2 National AIDS Control Council (NACC), Nairobi, Kenya; 3 University of Manitoba/University of Nairobi-SWOP Clinics, Nairobi, Kenya; 4 National AIDS and STIs Control Program (NASCOP), Nairobi, Kenya; 5 International AIDS Vaccine Initiative, Nairobi, Kenya; 6 World Health Organization, Geneva, Switzerland; University of Missouri-Kansas City, UNITED STATES

## Abstract

**Background:**

The MSM population in Kenya contributes to 15% of HIV incidence. This calls for innovative HIV prevention interventions. Pre-exposure prophylaxis (PrEP) has been efficacious in preventing HIV among MSM in trials. There is limited data on the willingness to take daily oral PrEP in sub-Sahara Africa. PrEP has not been approved for routine use in most countries globally. This study aimed to document the willingness to take PrEP and barriers to uptake and adherence to PrEP in Kenya. The findings will inform the design of a PrEP delivery program as part of the routine HIV combination prevention.

**Methods:**

Eighty MSM were recruited in 2 Counties in December 2013. Quantitative data on sexual behaviour and willingness to take PrEP were collected using semi-structured interviews and analysed using SPSS. Qualitative data on knowledge of PrEP, motivators and barriers to uptake and adherence to PrEP were collected using in-depth interviews and FGDs and analysed using Nvivo. Analysis of data in willingness to take PrEP was conducted on the HIV negative participants (n = 55).

**Results:**

83% of MSM were willing to take daily oral HIV PrEP. Willingness to take PrEP was higher among the bi-sexual and younger men. Motivators for taking PrEP were the need to stay HIV negative and to protect their partners. History of poor medication adherence, fear of side effects and HIV stigma were identified as potential barriers to adherence. Participants were willing to buy PrEP at a subsidized price.

**Conclusions:**

There is willingness to take PrEP among MSM in Kenya and there is need to invest in targeted education and messaging on PrEP to enhance adherence, proper use and reduce stigma in the general population and among policy makers.

## Introduction

Men who have sex with men (MSM) in Kenya bear a disproportionate HIV burden with a HIV prevalence of 18.2% compared to the national average prevalence of 6% [1–4]. According to the Kenya Modes of Transmission Survey (2009), an estimated 15.2% of all new HIV infections in Kenya occurred among the MSM population. This HIV incidence rate among MSM varied significantly across the regions; ranging from 6% to 20.5% in different regions [5]. As a bridging population, the MSM community has also been documented as key in driving the HIV epidemic due to concurrent heterosexual relations in the general population. There is need for innovative and targeted prevention interventions to ensure effective reduction on HIV amongst MSM. The success of such interventions should in turn result in an overall reduction of HIV transmission rate across all categories of key and vulnerable populations in Kenya and in the region.

HIV interventions that target MSM and all vulnerable populations in the national HIV strategic plans[6, 7]. Kenya developed the Prevention Roadmap, a strategy for reducing new HIV infections by 2030 [7, 8]. This Roadmap stipulates the high impact, evidence-based interventions that are targeted to priority populations and geographical locations that record high HIV incidence and prevalence. PrEP is the use of an antiretroviral drug by HIV negative people to prevent the acquisition of HIV infection. The efficacy of PrEP has been established in randomized control trials globally [9–12]. The effectiveness of PrEP in these studies has been closely linked to willingness to take PrEP, uptake and adherence, as two trials failed to produce evidence of effectiveness, largely as a result of poor adherence [13, 14]. It is important to know if people at increased risk of HIV infection who could potentially benefit from PrEP would actually be willing to take it and adhere as needed to receive the prevention benefit. It is also important to know what is required to deliver PrEP safely and effectively to achieve the desired prevention benefit at the least possible cost.

There is some evidence on the willingness to take PrEP among MSM in Kenya. The evidence is however in limited geographic locations and sample size [15, 16]. Evidence on the willingness to take PrEP among MSM is crucial in informing policy makers and actors in HIV prevention as they: design and implement PrEP demonstration projects and in addressing gaps in adherence to PrEP during scale up of PrEP as part of HIV combination prevention. This study was conducted before the implementation of demonstration project that aims to provide evidence on the deliverability of PrEP to female sex workers, men who have sex with men and young women in Kenya. This paper presents the findings of a cross sectional feasibility study that aimed to determine the willingness of MSM to take daily oral PrEP to prevent HIV infection and characterize the HIV risk among MSM. The study was conducted between December 2013 and April 2014 and applied mixed methods in data collection and analysis.

## Methods

### Study design

This cross sectional study was designed to provide evidence of the willingness to take daily oral PrEP among MSM populations in Kenya.

### Ethics statement

This study was carried out in accordance with the Kenya Government guidelines for research with human participants and ethical clearance was obtained from the Kenya Medical Research Institute Ethics Review Committee (NON-SSC Protocol Number 405)[17]. Investigators also ensured that the study was conducted in accordance with the best practices in conducting research with MSM communities[18]. MSM communities in the study sites were engaged in all the stages of this study i.e. design, community sensitization, recruitment and dissemination. Participants were required to provide written informed consent before participating in the study and they were informed that they could decline or withdraw from the study at any point. All data were de-identified and anonymized using unique identifiers at data entry and transcription. Data that were collected as part of this project, including notes from and recorded interviews can only be accessed by the research team in charge of analysis in a password protected electronic database. The entire research team received training on research ethics before the study began.

### Recruitment of participants

Since same sex is criminalized in Kenya [19], and therefore MSM are a hidden population. The following assumptions were therefore made: it would be difficult to access and enrol participants into the study and construction of a sampling frame would be challenging. Participants were therefore consecutively recruited using the respondent driven sampling (snow ball sampling) in Kisumu County at the LVCT Health HIV Testing and Counselling site and in the offices of a youth led organization that caters for HIV positive MSM and transgender sex workers. MSM in Nairobi County were purposively recruited from the membership of the Kenyan national LGBTI umbrella body. The recruitment sites were purposively selected based on advice from the MSM community stakeholders. Participants were eligible to participate in the study if they could communicate in English or Kiswahili, were willing to volunteer as participants. All participants were interviewed over a period of one month (December 2013). This cross sectional study was conducted to inform the design a PrEP demonstration project in Kenya and determine feasibility of implementing the demonstration project. This study was therefore not powered to demonstrate generalizable willingness to take PrEP among Kenyan MSM.

### Data collection

Participants completed self-administered questionnaires to collect data on demographics, HIV characteristics, sexual behaviour, history of STIs, knowledge of HIV, self-perceptions of HIV risk and willingness to take oral daily PrEP. A subset of HIV negative MSM who completed the self-administered questionnaires were systematically (every 8^th^ participant) selected to participate in an in-depth interview. Focus group discussions (FGD) of between 8 and 10 people were held with the HIV negative MSM in each County for purposes of triangulating the responses from the self-administered questionnaires and in-depth interviews. FGD participants were systematically (every 8^th^ person) selected from the sampling frame of participants in the in-depth interviews in Nairobi and those who completed the questionnaires in Kisumu County.

The in-depth interviews (IDIs) explored topics on knowledge of PrEP, possible barriers to effective use, regular HIV testing and concerns about taking daily PrEP pills. Focus group discussions explored topics on knowledge of HIV risk behaviour and practices, perceptions of HIV risk, factors influencing reporting sexual behaviour, knowledge of PrEP, factors influencing choice of prevention, practices and preferences of HIV prevention, willingness to have an HIV test and taking of a daily oral PrEP dose, motivators and perceived barriers to uptake of counselling and adherence to PrEP. Data were collected in both English and Swahili.

### Primary outcome

The primary outcome of this study was willingness to take daily oral PrEP to prevent HIV infection. Participants were required to answer “yes” or “no” to the question “If there was a daily pill that people would take to prevent HIV infection, would you take it?” The secondary outcome was to study the associations between the MSM sexual behaviour and practices and willingness to take daily oral PrEP.

### Data analysis

All quantitative data were analysed using the Statistical Package for Social Scientists [IBM Corp. Released 2013. IBM SPSS Statistics for Windows, Version 22.0. Armonk, NY: IBM Corp]. Descriptive statistics were used to describe the characteristics of the participants and their willingness to take daily oral PrEP. The Pearsons Chi Square and Fishers Exact tests were applied to determine associations between the participants’ characteristics and their willingness to take PrEP. Binary logistic regression modelling was used to determine the strength of any significant associations. A p value of less than 0.05 was considered statistically significant and missing data were omitted from the analysis.

Qualitative data from the FGDs and in-depth interviews were coded in NVIVO version 10 (NVivo qualitative data analysis software; QSR International Pty Ltd. Version 10, 2012.). A framework analytical approach, guided by the study objectives, was developed and used to identify the emerging themes and concepts from the transcripts. In-depth interviews explored topics about: Knowledge of PrEP among MSM, possible barriers to effective use of PrEP, regular HIV testing and concerns about PrEP. FGDs explored discussions about: practices and behaviour that will influence effective use of PrEP, knowledge of PrEP and motivators for taking PrEP. Themes that emerged and used during analysis were: perceived barriers to effective use of PrEP, concerns about PrEP, settings are appropriate for PrEP delivery, strategies to optimize effective use of PrEP and the need for regular HIV testing. Analysed data was presented in a descriptive manner and selected quotes illustrating common themes included in the text.

## Results

### Demographic characteristics

A total of 80 MSM completed the self-administered questionnaire in Kisumu and Nairobi Counties. Out of the 80 MSM participants, 68.8% reported being HIV negative (n = 55). The analyses in the rest of this paper will focus on the HIV negative MSM. The participants’ median age was 24.9 years (IQR = 5 years), with the majority (89%) being in the age-group 18 to 29 years.

About three quarters of the participants (n = 38) resided in urban settings, 16% (n = 8) in slum dwellings and 10% (n = 5) in rural settings. Of this total population 18.2% resided with their sexual partner and 41.8% resided alone. Nineteen (34.5%) participants had recently completed secondary education and sixty percent had attended tertiary institutions of learning (n = 33). Twenty four percent of the MSM had an income from self-employment and 21.6% (n = 11) were casual workers. Six participants (11.8%) identified themselves as sex workers. Preference for anal sex was reported by 51.9% of the MSM compared to vaginal or oral was reported by 25% (n = 13) and 17.3% (n = 9), respectively; although this was not categorized as insertive or receptive.

### Sexual orientation and characteristics

About half (49.1.8%) of the MSM surveyed identified themselves as being exclusively homosexual (only engaged in sex with men), while the rest identified themselves as being bisexual (engaged in sex with both men and women). About half of the MSM were single (n = 26); 34.5% (n = 19) were in a relationship with a man and one was married or in a civil union or relationship with a woman. All single MSM in this study reported having casual sexual partners. Although 23.6% (n = 13) reported having steady and committed partners, 55% (n = 11) single MSM and 30% (n = 6) reported having had between 3 to 5 other male sexual partners in the last 1 year. More MSM aged between 24 to 29 years reported having multiple sexual partners compared to those below 24 years, although the difference was not significant (χ^2^ = 15.23; d.f = 14; p = 0.242).

### Condom use among HIV negative MSM

As illustrated in Table 1, most of the HIV negative MSM reported using condoms during sexual intercourse with either any other and their main sexual partners.

**Table 1:** Reported condom use among HIV negative participants (n = 55).

	Association between age categories and frequency of condom use with any sexual partner in last 12 months (n = 55)			
	Never	Sometimes	Always	Total (n)
18–23 years	1	7	16	24
24–29 years	0	8	16	24
= >30 years	0	2	4	6
	Association between age categories and use of condom with main sex partner (n = 55)			
	Never	Sometimes	Always	Total (n)
18–23 years	3	7	14	24
24–29 years	0	11	13	24
= >30 years	1	2	3	6

1. Test statistics for association between age categories and frequency of condom use with any sexual partner: χ^2^ = 1.324; d.f = 4; p = 0.857; Fisher’s exact test = 2.014; p>0.999

2. Test statistics for Association between age categories and use of condom with main sex partner: χ^2^ = 4.313; d.f = 4; p = 0.365; Fisher’s exact test = 4.792; p = 0.326

3. Data from one respondent was dropped from the analysis due to incomplete responses.

### Willingness to take daily oral HIV PrEP

Analysis on willingness to take daily PrEP was assessed in only those who reported to be HIV negative (n = 55) based on their last test. Eighty three percent (83.3%; 95% CI: 71.3% to 91%) of the HIV negative MSM expressed their willingness to take the daily PrEP pill if it was made available to them. Table 2 illustrates the association between willingness to take daily oral PrEP and demographic and sexual practices such as condom use and sexual assertiveness. Younger MSMs—aged below 30 years—reported higher levels of willingness to take oral PrEP compared to the older MSMs.

**Table 2:** Association between willingness to take daily oral PrEP and selected baseline characteristics among the MSM (n = 55).

	Willing to take daily oral PrEP	Not willing to take daily oral PrEP	Total	P value
Level of education attained				0.470
Some secondary	2(66.7%)	1(33.3%)	3 (100%)	
Secondary completed	17 (89.5%)	2(10.5%)	19(100%)	
College/ University	26(81.2%)	6 (18.8%)	32(100%)	
Relationship/Marital status				0.157
Single	23 (88.5%)	3 (11.5%)	26(100%)	
Married/ civil union (Woman)	0	1	1	
Have a boyfriend	15 (83.3%)	3 (16.7%)	18(100%)	
Have a girlfriend	1(50.0%)	1(50.0%)	2(100%)	
Several partners	6 (85.7%)	1(14.3%)	7(100%)	
Age groups				0.616
Less than or equal to 22 years	13(92.9%)	1(7.1%)	14 (100%)	
23–27 years	21(77.8%)	6(22.2%)	27 (100%)	
28–32 years	7(77.8%)	2(22.2%)	9 (100%)	
More than or equal to 33 years	3(100.0%)	0	3 (100%)	
Sexual assertiveness with main partner				0.786
Not possible	8(80.0%)	2(20.0%)	10 (100%)	
Difficult but can be done	20(83.3%)	4(16.7%)	24 (100%)	
Easy	16(88.9%)	2(11.1%)	18 (100%)	
Sexual assertiveness with other men				0.203
Not possible	6(66.7%)	3(33.3%)	9 (100%)	
Difficult but can be done	22(91.7%)	2(8.3%)	24 (100%)	
Easy	17(81.0%)	4(19.0%)	21 (100%)	
Sexual orientation				0.025
Homo sexual	20 (74.1%)	7 (25.9%)	27 (100%)	
Bisexual	25 (96.2%)	1 (3.8%)	26 (100%)	
Condom use with main sexual partner				0.842
Never	3	0	3(100%)	
Sometimes	16(80%)	4(20%)	20(100%)	
Always	26(84%)	5(16%)	31(100%)	

Missing data were omitted from the analysis

### Knowledge of PrEP

A few of the participants reported to have heard about PrEP through their social networks since Truvada^™^ is not registered for PrEP in Kenya. This was demonstrated in these comments by MSM who participated in an in-depth interview: “it’s only available in South Africa” and “….it is targeting the discordant couples. So I don’t know if I heard it wrongly or if am on safe track.”

The lack if in-depth knowledge of PrEP can be attributed to the fact that PrEP is a new concept in Kenya and it still has not been approved for HIV prevention.

Other participants had heard of PrEP but from other countries as one FGD participant commented:

“How I wish this thing was initiated 100 years ago! Now we just get it because of the results that have been achieved from other experiences. I feel that if I were to go for this thing, [I] am convinced of the benefits, am convinced of the side effects, I’m convinced of the cost effectiveness in it. Then there nobody will remind me to take it. I will be motivated to take it.”

Others were dubious about the benefits of PrEP, and expressed their willingness to take it more reservedly. Despite their caution about taking daily oral PrEP, the participants stated that they would be willing to enrol into a PrEP program since any method that reduces their risk of HIV was seen as important. They indicated that they would overcome their hesitancy to take PrEP if they had adequate information and reassurance on the safety of PrEP. “If am sure it is going to work, it will motivate me to take it.” (FGD Participant). Concerns about side-effects were prominent. As one participant summarized;

“Me, I will not use it, because I don’t know if it will cause some harm in my body.” (MSM_FGD Participant)

### Willingness to take PrEP and Sexual orientation

MSM who self-identified as being bi-sexual were 8.75 times more likely to be willing to take oral PrEP (OR = 8.75, SE = 1.1, p = 0.051) compared to MSM of exclusive homosexual orientation. Logistic regression on sexual orientation, predicted an 85% effect of sexual orientation on the willingness to take PrEP. The main motivation for wanting to take PrEP was the desire to remain HIV-negative. “Just like [identity withheld] said, if you have it then you are, like, safe. If you don’t have, then you are unsafe.” (FGD Participant)

### Sexual behaviour and practices

Lack of sexual assertiveness was predictive of willingness to take HIV PrEP. Of the 19 participants who reported lack of sexual assertiveness, 80% lacked sexual assertiveness with their main male sexual partner and 67% lacked assertiveness with male casual sexual partners. Participants who lacked sexual assertiveness were more willing to take oral PrEP compared to those who reported being sexually assertive. This observation was however not statistically significant as illustrated in Table 2.

### Barriers for effective use of PrEP

A number of participants in the FGDs reported a history of not completing courses of medicine. This pattern became particularly pronounced when paired with a general dislike of medicine taking as stated by a participant in an in-depth interview: “I don’t like to take medicine. Normally I take for a short while and then when I start to feel better I stop taking”. A number of participants also predicted that they would only take PrEP on the days that they were exposed. This evinces the need for education on how PrEP works to prevent infection. FGD participants cited alcohol abuse and binge drinking as potential barriers to PrEP adherence. Discussants highlighted that binge drinking would lead to them forgetting to take PrEP on a daily basis.

Various ways to achieve good adherence were discussed during the FGDs. Since most of the participants reported that they experience challenges adhering to medication, the usefulness of frequent reminders was found to be preferable. Other popular ideas of enhancing adherence included peer-to-peer education to effectively spread information; counselling and support groups for MSM on PrEP. Group meetings were discussed as being of particular value because they could also provide a forum to discuss challenges, develop solutions, and educate men on how to take PrEP properly. Participants acknowledged that in order to ensure adherence to PrEP, broader community-based interventions—that would raise awareness and decrease stigma—are required. Ideas for educating the broader community included informational campaigns on billboards, television, and radio.

### Concerns about PrEP

Generally, participants simply wanted more information about PrEP: “What I need to know is what it does in the body, some information about it, if it protects one from HIV/AIDS and STIs, if it fattens, its advantages and disadvantages and after knowing all that I’ll be comfortable.” (IDI_Participant). Majority of the participants wanted information about PrEP’s interaction with alcohol.

Many participants were concerned about PrEP use being discovered and the social costs associated with others knowing that they were taking ARVs. One man explained, “As I said, I worry first of all, I worry of stigma, because some people with think of PrEP is an ARV, so they will say that this person is [HIV] positive.” The value of social separation from medicine used to treat HIV was seen by many as paramount.

Participants who wanted PrEP packaged differently feared that peers would begin “classifying you as HIV positive.” One sex worker felt that he would tell his regular partner but not his clients about taking PrEP.

Of particular concern were MSM who are married to women: “They are not safe at all. If you are MSM that you are married and your wife have been hearing stories but you have never been caught that you sleep with other men, but you don’t want to sleep with her, then you want to PrEP yourself, no way.” (IDI_Participant).

The high potential for creating disharmonious or acrimonious tension within relationships may explain why many participants wanted to conceal that they were taking PrEP and hide the pills themselves.

Some participants were willing to buy PrEP at a maximum price of 100 shillings (Approximately 1 USD) for a month’s supply. Others felt that the price of PrEP should be equivalent to the cost of condoms and one man questioned why he should consider paying for PrEP if condoms are available to him at no cost. A few participants felt that people would value and trust PrEP more if they had to pay for it.

He explained: “I would go for one shilling per tablet and it would also make people more responsible because if it is free now people will just leave the other preventive measures. But if someone feels they are buying it, then they will be responsible”

Preferred locations for getting PrEP included local shops, pharmacies, kiosks, MSM-friendly clinics and through government-run hospitals and clinics and available only through prescription.

A general sense of uncertainty permeated the discussions around PrEP’s newness. When one interview reassured FGD participants that, “[PrEP] has been used in other countries. Now they want to introduce it in Kenya” one respondent seemed reassured but clarified, “So it is not research they are coming to do with us?”

Some people were not yet willing to trust the effectiveness of PrEP but understood that research is necessary. “The same people who made PREP and the same ones who made condoms and those people are human beings and human beings are never perfect…”(IDI_Participant)

### Deliverability of HIV PrEP

#### Regular HIV testing

While a large group of the men interviewed reported no problem returning for the required regular HIV tests, some men did raise certain concerns and barriers. Some were based on the practicalities like the inconvenience of making an additional trip to the clinic, distance, potential shortages in testing supplies at clinics, and the friendliness of staff. Some however felt that after initiating PrEP, they would return to the “clinic to see if [PrEP] will work, but after that, if it works, I will rarely visit clinic, will just continue to use the medicine, but will rarely visit clinic”

A noteworthy observation is that the in-depth interviews yielded similar responses as the FGDs across all the discussion topics.

## Discussion

This study is among the first qualitative research on willingness to take PrEP among MSM in Kenya. Findings from this study respond to the call for additional research on which MSM sub-populations to focus on for HIV prevention using PrEP provision [20]. These findings also have implications for: design of PrEP demonstration projects; development of HIV combination prevention strategies and formulation of policy for delivering PrEP as part of combination prevention.

The high levels of reported willingness to take the daily oral PrEP pill (83%) among the MSM in this study are consistent with the findings of an RCT by Mutua et al., which documented high levels of acceptance of PrEP (83%) among MSM; the authors also reported high adherence of over 80% [15] among MSM who were in the daily PrEP arm. A multi-country study by Eisingerich et al. reported slightly lower levels of willingness to take PrEP among Kenyan MSM at approximately 60% [16]. Participants in our study further stated that they would be willing to take daily oral PrEP if the cost was subsidized to a price equivalent to that of condoms. These finding are consistent with those of Mutua et al. who documented that acceptance of PrEP was tied to the price [15].

Of particular interest is the high willingness among exclusive MSM to take PrEP. As shown in a number of studies, exclusive MSM who participate in insertive or penetrative anal intercourse (AI) report low condom use and PrEP is probably perceived as an additional layer of protection in situations where condoms are not used. Studies have reported that inconsistent condom use among MSM is due to: diminished sexual pleasure; condoms being perceived as cumbersome due to the time taken to wear them and; the promise of extra money for male sex workers if they engage in condomless sex [21–23]. PrEP is probably perceived beneficial in prevention during episodes where condoms are not used during AI. This finding has crucial program implications since it demonstrates the need for enhanced counselling and education on HIV transmission while delivering PrEP to reduce risk compensation and promote the use of condoms and water based lubricants during AI as pointed out by Geibel et al. [24]. The likelihood of younger MSM to be more likely to be willing to take oral PrEP is consistent with other studies which document them to be more sexually active than the older ones[25]. This is the same age-group that reported to have higher levels of HIV risky behaviour such as partial condom use during anal intercourse and multiple sexual partners. This is a potential age cohort to focus on for PrEP interventions.

This study documented concerns from MSM that would deter adherence to daily oral PrEP. Van der Elst et al. reported that despite the side effects that participants experienced while taking PrEP, this did not affect their adherence since the side effects subsided after sometime. Those who experienced stigma from family and partners coped by concealing their pills or resorted to lying about the pills [26]. Our study provided more data on the negative effects of HIV related stigma, which may negatively affect adherence to PrEP. Poor adherence may have negative effects in the HIV epidemic and prevention efforts if not monitored effectively. This calls for closer monitoring of the use of PrEP in real life settings. Since stigma was indicated as a possible reason for non-adherence, there will be need for the wider public to be educated on PrEP to reduce the stigma that is associated with HIV. Our findings indicate the demand for more knowledge and information on PrEP.

Bisexual men in this study reported significantly higher levels of willingness to take PrEP compared to those who identified themselves as exclusive MSM. This is probably because MSM in bi-sexual relationships perceive themselves to be at higher risk and hence the higher levels of willingness to add PrEP to their prevention arsenal [16]. A number of studies have documented that MSM who are in concurrent sexual relationships with men and women are exposed to sexual practices that expose them to HIV infection such as inconsistent condom use due to the long term attachments and intimacy with concurrent sexual partners [23, 27]. Moreover, Baral et al. documented that bi-sexual men were less likely to use condoms with both committed and casual female sexual partners [28]. These HIV risky sexual practices may have a role to play in influencing higher willingness to take PrEP among bi-sexual MSM. Given the high prevalence of HIV among bi-sexual MSM in Kenya and sub-Saharan Africa [5, 19, 29], this is a strategic population to target with PrEP as part of combination prevention.

Our study found a significant association between willingness to take daily oral PrEP and lack of sexual assertiveness. Lack of sexual assertiveness with male sexual partners may be due to a number of factors such as: involvement in sex work, poverty and imbalanced power relations with sexual partners (especially in cross-generational sexual relationships). Okal et al. further reported that in relationships where there is considerable power imbalance, ability to negotiate condom use is compromised since safe sex practices are often only be feasible if the perpetrator opted for safe protection during anal sex [21]. MSM who sell sex may also find themselves in situations where they may not be able to reject anal sex due to poverty and the power that their clients and law enforcement agents have over them as documented by Okanlawon et al.[22]. These factors that compromise sexual assertiveness may contribute to high willingness to take PrEP as an additional layer of protection.

Although our study did not find a significant association between condom use and willingness to take daily oral PrEP, it is important to target MSM in combination prevention interventions that include PrEP. This study revealed that 33% of the MSM reported using condoms “sometimes” during anal sex with their main sexual partner and 35% with other male casual sexual partners. Our findings are consistent with recent research that showed similar patterns of inconsistent condom use among MSM in Kenya [20, 30]. This indicates the level of HIV transmission risk that MSM are exposed to by engaging in condom less anal intercourse [31]. PrEP would be a feasible alternative for HIV prevention for MSM in situations where they may not be in a position to negotiate for condom use such as: sex work and imbalanced relationships [21, 22].

This study had a number of limitations. First, the sample size of participants was small and hence the findings on willingness to take PrEP may not be generalizable to the entire MSM population. Second, the HIV status of the participants was self-reported and was not ascertained during the study. Third, this study aimed to assess the willingness to take PrEP—within a broader demonstration project. This findings are crucial for designing PrEP programs and strategies. The experiences associated with long-term use of PrEP will be documented in the subsequent demonstration project.

## Conclusion

There is need for the inclusion of oral PrEP as part of the biomedical interventions to prevent HIV transmission. This study adds to the body of knowledge data on willingness to take daily oral PrEP for HIV prevention. This study concludes that there is willingness among MSM in Kenya to take PrEP and that there is need to invest in education on PrEP as an approach in HIV prevention to enhance adherence, proper use and reduce stigma. This study recommends the following:

1. Actors in HIV prevention need to work with the MSM community—that has demonstrated willingness to take PrEP- in demonstration projects so as to understand how PrEP can be cost effectively delivered as part of combination prevention, and; how to tailor messages and interventions that promote adherence to PrEP among MSM in Kenya.
2. Effective messaging and education need to be undertaken to: ensure correct knowledge of PrEP; eliminate misconceptions on PrEP and emphasize its importance and correct use as part of the existing HIV prevention strategies. These messages need to be tailored for the different sub-groups of key populations and also general population and policy makers.
